# Anemarsaponin BII inhibits the activity of CYP3A4, 2D6, and 2E1 with human liver microsomes

**DOI:** 10.1080/13880209.2020.1835996

**Published:** 2020-10-26

**Authors:** Mingwei Wang, Wei Jiang, Juan Zhou, Xiujuan Xue, Changlong Yin

**Affiliations:** Department of Pediatrics, Yidu Central Hospital of Weifang, Weifang, China

**Keywords:** CYP450 enzymes, CYP2D6, CYP2E1, drug–drug interaction

## Abstract

**Context:**

Anemarsaponin BII is one of the most active saponins isolated from *Anemarrhena asphodeloides* Bunge (Asparagaceae), a commonly used Chinese traditional paediatric medicine.

**Objective:**

This study investigates the effects of anemarsaponin BII on the activity of CYP450s to provide more guidance for the clinical use of anemarsaponin BII.

**Materials and methods:**

Using various diagnostic substrates, the effects of a fixed concentration of anemarsaponin BII (100 μM) on the activity of eight main isoforms of CYP450s (CYP1A2, 2A6, 3A4, 2C8, 2C9, 2C19, 2D6 and 2E1) was first studied with pooled human liver microsomes (HLMs). Then, dose-dependent (0, 2.5, 5, 10, 25, 50 and 100 μM anemarsaponin BII) and time-dependent (0, 5, 10, 15 and 30 min) experiments were performed to obtain corresponding kinetic parameters.

**Results:**

Anemarsaponin BII showed significant inhibitory effects on the activity of CYP3A4, 2D6 and 2E1 with the IC_50_ values of 13.67, 16.26 and 19.72 μM. Anemarsaponin BII acted as a non-competitive inhibitor of CYP3A4 with the *K_I_* value of 6.72 μM and competitive inhibitors of CYP2D6 and 2E1 with the *K_I_* values of 8.26 and 9.82 μM, respectively. Additionally, the inhibition of CYP3A4 was revealed to be time-dependent with the *K_I_* value of 4.88 μM and the *K_inact_* value of 0.053/min.

**Conclusions:**

The inhibitory effect of anemarsaponin BII on the activity of CYP3A4, 2D6 and 2E1 indicated the potential drug–drug interaction between anemarsaponin BII and drugs metabolized by these CYP450s. Further *in vivo* experiments are needed to validate the potential drug–drug interactions.

## Introduction

Cytochrome P450 enzymes (CYP450s) comprised a superfamily of hemoproteins that participate in various oxidative reactions and play an essential role in the metabolism of xenobiotics (Manikandan and Nagini 2018). Over 50 isozymes have been identified in humans, of which CYP1A2, 2A6, 3A4, 2C8, 2C9, 2C19, 2D6 and 2E1 are the most common types (Sychev et al. 2018). The activity of CYP450s is a key factor that is responsible for the majority of the drug–drug interaction. For example, cannabidiol and clobazam are commonly used drugs in paediatric refractory epilepsy, the conjunctive use of these two drugs induced side effects through the CYP450 pathway (Geffrey et al. 2015). Several studies have reported various drugs exerted inhibitory or inductive effects on the activity of CYP450s, such as pinocembrin, chrysin, apigenin, galangin and honokiol (Wang et al. 2018; Bojic et al. 2019). Declaiming the activity of CYP450 under different drug administrations is of great significance for the clinical use of drugs, especially for the potential co-administrated drugs.

*Anemarrhena asphodeloides* Bunge (Asparagaceae) is traditional medicine in China and many other Asian countries that has been widely administrated for thousands of years. *Anemarrhena asphodeloides* was reported to possess anti-inflammatory, immune-stimulating and anti-neuroinflammatory effects (Ji et al. 2019). In paediatric use, *A. asphodeloides* is employed for the treatment of paediatric epilepsy, fever, coughing and allergies due to its properties of removing pathogenic heat (Park et al. 2008; Wang et al. 2014). Moreover, *A. asphodeloides* was reported to eliminate urinary protein, which makes it useful for the treatment of acute nephritis in children. There is a variety of compounds in *A. asphodeloides*, such as saponins, flavonoids, phenylpropanoids, and alkaloids, among which saponins are the main ingredients responsible for the biological function of *A. asphodeloides* (Wang et al. 2014; Ji et al. 2017; Xia et al. 2017). Anemarsaponin BII is one of the most active saponins isolated from *A. asphodeloides* that was found in high concentrations (Kim et al. 2009; Zhao et al. 2016). Commonly in traditional Chinese medicine, co-administration of various herbs can significantly improve therapeutic efficiency. Therefore, investigating the effect of anemarsaponin BII on the activity of CYP450s can provide more guidance for its clinical application and combination with other drugs.

The interaction between anemarsaponin BII and eight CYP450 isoforms was investigated in human liver microsomes in this research, in order to reveal the *in vitro* effects of anemarsaponin BII on the activity of CYP450s.

## Materials and methods

### Chemicals

Anemarsaponin BII (≥98%) and testosterone (≥98%) were purchased from the National Institute for the Control of Pharmaceutical and Biological Products (Beijing, China). d-Glucose-6-phosphate, glucose-6-phosphate dehydrogenase, corticosterone (≥98%), NADP^+^, phenacetin (≥98%), acetaminophen (≥98%), 4-hydroxymephenytoin (≥98%), 7-hydroxycoumarin (≥98%), 4′-hydroxydiclofenac (≥98%), sulfaphenazole (≥98%), quinidine (≥98%), tranylcypromine (≥98%), chlorzoxazone (≥98%), 6-hydroxychlorzoxazone (≥98%), paclitaxel (≥98%), 6β-hydroxytestosterone (≥98%), clomethiazole (≥98%) and furafylline (≥98%) were obtained from Sigma Chemical Co. Montelukast (≥98%) was obtained from Beijing Aleznova Pharmaceutical (Beijing, China). Coumarin (≥98%), diclofenac (≥98%), dextromethorphan (≥98%), and ketoconazole (≥98%) were purchased from ICN Biomedicals. Pooled HLMs were purchased from BD Biosciences Discovery Labware. All other reagents and solvents were of analytical reagent grade.

### Assay with human liver microsomes

The activity of CYP450s was evaluated by the marker reactions with specific substrates in human liver microsomes (HLMs). The reaction conditions are summarized in Table 1 according to previous studies. The concentration of anemarsaponin BII was 100 μM with the following concentration of specific inhibitors: 10 μM furafylline for CYP1A2, 10 μM tranylcypromine for CYP2A6, 1 μM ketoconazole for CYP3A4, 5 μM montelukast for CYP2C8, 10 μM sulfaphenazole for CYP2C9, 50 μM tranylcypromine for CYP2C19, 10 μM quinidine for CYP2D6, 50 μM clomethiazole for CYP2E1. 200 μL incubation system included 100 mM potassium phosphate buffer (pH 7.4), NADPH generating system (1 mM NADP^+^, 10 mM glucose-6-phosphate, 1 U/mL of glucose-6-phosphate dehydrogenase, and 4 mM MgCl_2_), the appropriate concentration of HLMs, a corresponding probe substrate and anemarsaponin BII (or positive inhibitor for different probe reactions). The HADPH-generating system was added before the incubation to preincubate for 3 min at 37 °C. The reaction was terminated with 100 μL acetonitrile (10% trichloroacetic acid for CYP2A6). Then, the mixture was centrifuged at 12,000 rpm for 10 min, and an aliquot (50 μL) of supernatant was transferred for HPLC analysis with the help of the Agilent 1260 series instrument with DAD and FLD detector according to previous studies. The HPLC conditions for analysis of corresponding metabolites of CYP isoforms are summarized in Table 2 based on previous studies (Lang et al. 2017; Zhang et al. 2017).

**Table 1.** Isoforms tested, marker reactions, incubation conditions, and *K_m_* used in the inhibition study.

CYPs	Marker reactions	Substrate concentration (μM)	Protein concentration (mg/mL)	Incubation time (min)	Estimated *K_m_* (μM)
1A2	Phenacetin *O*-deethylation	40	0.2	30	48
3A4	Testosterone 6β-hydroxylation	50	0.5	10	53
2A6	Coumarin 7-hydroxylation	1.0	0.1	10	1.5
2E1	Chlorzoxazone 6-hydroxylation	120	0.4	30	126
2D6	Dextromethorphan *O*-demethylation	25	0.25	20	4.8
2C9	Diclofenac 4′-hydroxylation	10	0.3	10	13
2C19	S-Mephenytoin 4-hydroxylation	100	0.2	40	105
2C8	Paclitaxel 6α-hydroxylation	10	0.5	30	16

**Table 2.** HPLC conditions for the determination of corresponding metabolites of mentioned CYP isoforms.

CYP isoforms	Detection wavelength (nm)	Mobile phase gradient
1A2	245	Methanol:phosphate buffer (pH = 3.0) = 31:69
2A6	Fluo Ex/Em: 340/456	Acetonitrile:acetic acid (0.1%) = 35:65
3A4	254	Methanol:Water = 50:40; 0–15 min, 48% B–30% B; 15–20 min, 30%B–20% B
2C8	230	Methanol:water = 65:35
2C9	280	Acetonitrile (A):phosphate buffer (pH = 7.4, B) = 35:65; 0–9 min, 65% B–35% B
2C19	204	Methanol:potassium phosphate (pH 7.0) = 30:70
2D6	Fluo Ex/Em: 235/310	Acetonitrile:phosphate buffer (Ph = 3.0 ) = 25:75
2E1	287	Acetonitrile:acetic acid (0.5%) = 20:80, 1–10 min, 78% B–40% B

Ex: excitation; Em: emission: Fluo: fluorescence.

Incubations were performed in triplicate, and results were represented with the mean value. The final microsomal protein concentration and incubation times for the different probe reactions are summarized in Table 1 based on previous studies (Lang et al. 2017; Wang et al. 2019).

### Enzyme inhibition and kinetic studies

The effect of anemarsaponin BII on the activity of CYPs was evaluated in pooled human liver microsomes according to previous studies (Dong et al. 2018; Wang et al. 2019). The residual activity of eight CYP isoforms was first detected with 100 μM anemarsaponin BII in HLMs. Next, CYPs of which the activity was inhibited by anemarsaponin BII was incubated with 0, 2.5, 5, 10, 25, 50 and 100 μM anemarsaponin BII to explore the values of half inhibition concentration (IC_50_). Additionally, 0–50 μM anemarsaponin BII was incubated with various concentration of probe substrates (20–100 μM testosterone for CYP3A4, 10–50 μM dextromethorphan for CYP2D6, and 25–250 μM chlorzoxazone for CYP2E1) to obtain the values of *K_I_*.

### Time-dependent study

The effect of incubation time on the inhibition of anemarsaponin BII was evaluated with 20 μM anemarsaponin BII. HLMs were preincubated with anemarsaponin BII and the NADPH-generating system for 30 min at 37 °C. Then, an aliquot (20 μL) was transferred to another incubation tube (final volume 200 μL) containing an NADPH-generating system and probe substrates, of which final concentrations were approximate to *K_m_*. After incubating for 0, 5, 10, 15 and 30 min, the reaction was terminated with 100 μL acetonitrile internal standard mix and placed on ice. The metabolites were analyzed by HPLC. To obtain the values of *K_I_* and *K_inact_*, another incubation with a higher concentration of substrates (approximately 4-fold *K_m_* values) was performed in the presence of 0–50 μM anemarsaponin BII for 0, 5, 10, 15 and 30 min.

### Statistical analysis

The enzyme kinetic parameters for the probe reaction were estimated from the best fit line, using least-squares linear regression of the inverse substrate concentration versus the inverse velocity (Lineweaver–Burk plots), and the mean values were used to calculate *V_max_* and *K_m_*. Inhibition data from the experiments that were conducted using multiple compound concentrations were represented by Dixon plots, and inhibition constant (*K_I_*) values were calculated using non-linear regression according to the following equation:

$$\text{Competitive inhibition}：v=(V_{\max}S)/(K_{m}(1+I/K_{I})+S),$$

$$\text{Non}-\text{competitive inhibition}:v=\left(V_{max}\text{S}\right)/[K_{m}+\text{S}(\text{1}+I/{\text{K}}_{\text{I}}{\text{K}}_{\text{i}})]$$

where *I* is the concentration of the compound, *K_I_* is the inhibition constant, S is the concentration of the substrate, and *K_m_* is the substrate concentration at half the maximum velocity (*V_max_*) of the reaction. The mechanism of the inhibition was inspected using the Lineweaver–Burk plots and the enzyme inhibition models. The data were obtained from triplicate experiments and the data comparison was performed using Student’s *t*-test and performed using IBM SPSS statistics 20 (SPSS Inc.).

## Results

### Effect of anemarsaponin BII on the activity of CYP450s

**Figure 1.**
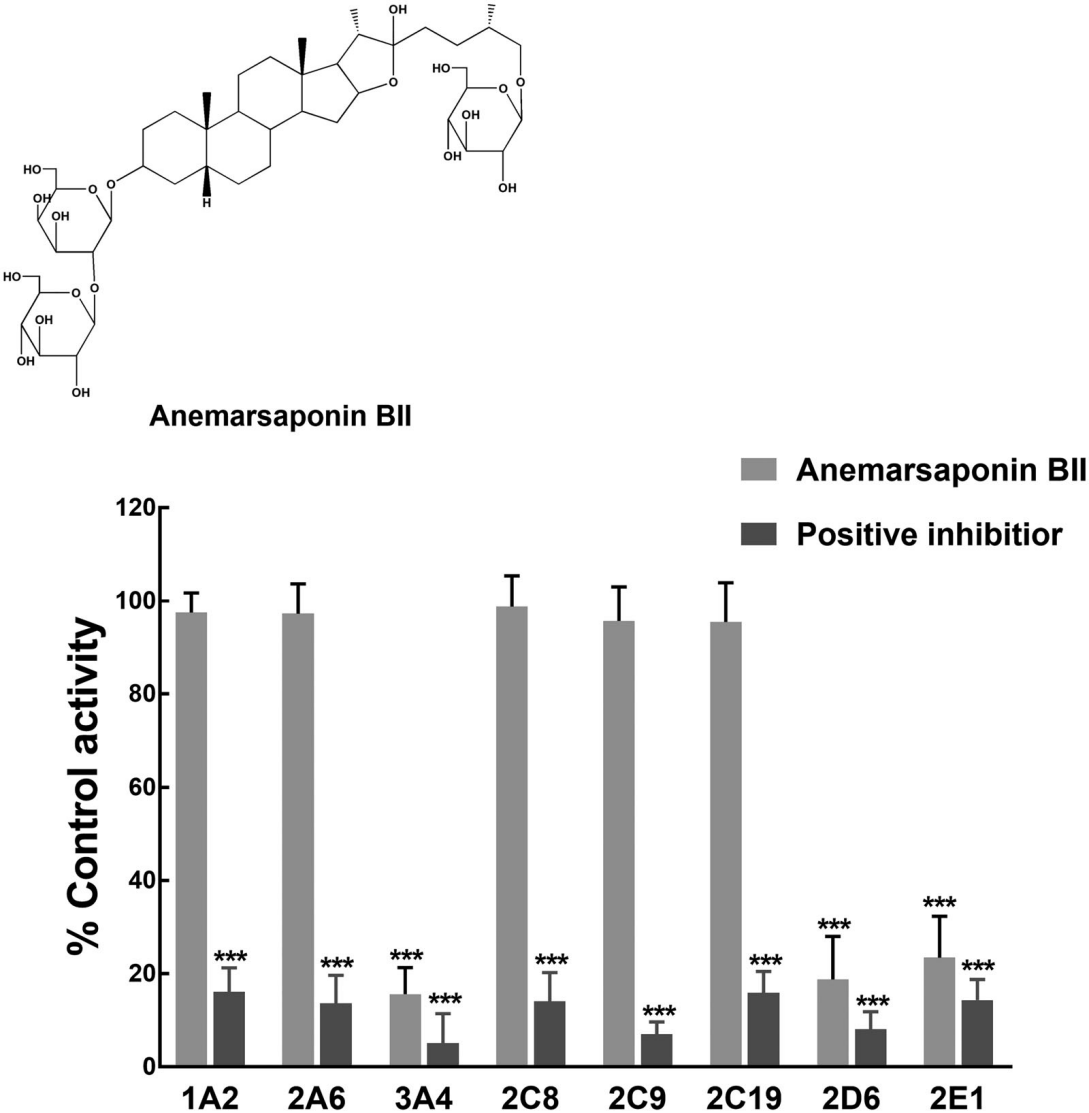
Effect of anemarsaponin BII and positive inhibitors on the activity of CYP450 enzymes in HLMs, including CYP1A2, 2A6, 3A4, 2C8, 2C9, 2C19, 2D6, and 2E1, relative the activity in untreated HLMs. ***p < 0.001. Data are obtained from the incubation with 100 μM anemarsaponin BII and various concentrations of positive inhibitors.

**Figure 2.**
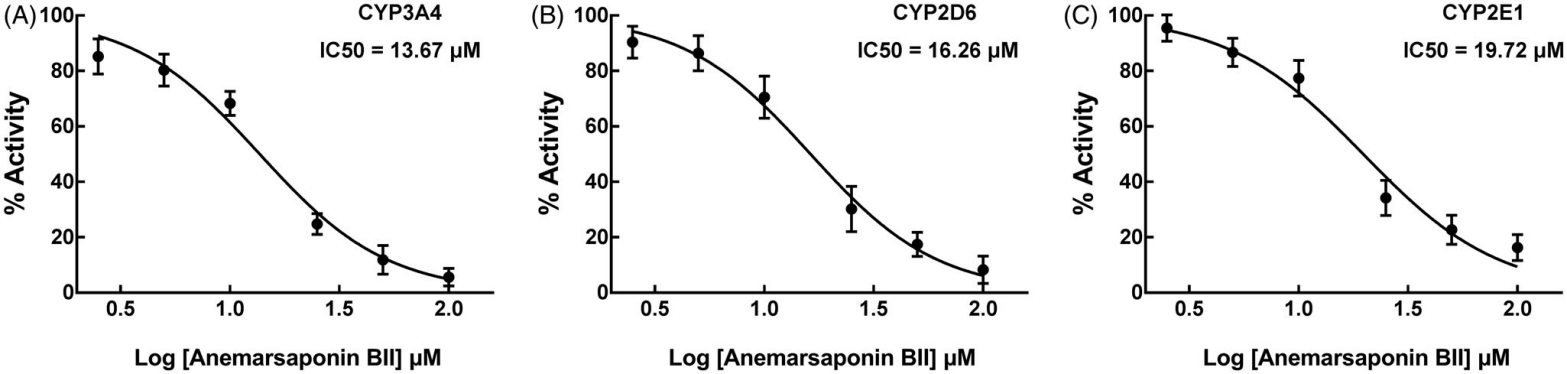
(A–C) Dose-dependent inhibition of CYP3A4, 2D6, and 2E1 by anemarsaponin BII.

Compared with untreated HLMs, the pre-treatment of positive inhibitors had a clearly significant effect on the activity of all CYP isoforms, and only the activity of CYP3A4, 2D6 and 2E1 was significantly inhibited by anemarsaponin BII. The activity of CYP3A4 was decreased to 15.65% and CYP2D6 and 2E1 decreased to 18.79 and 23.45%, respectively, in the presence of 100 μM anemarsaponin BII (Figure 1). While the inhibitory effects of anemarsaponin BII were weaker than that of specific inhibitors. In the presence of different concentrations of anemarsaponin BII, the inhibition of CYP3A4, 2D6, and 2E1 was found to be in a dose-dependent manner with the IC_50_ values of 13.67, 16.26 and 19.27 μM (Figure 2).

### The inhibition model of CYP3A4, 2D6 and 2E1 by anemarsaponin BII

**Figure 3.**
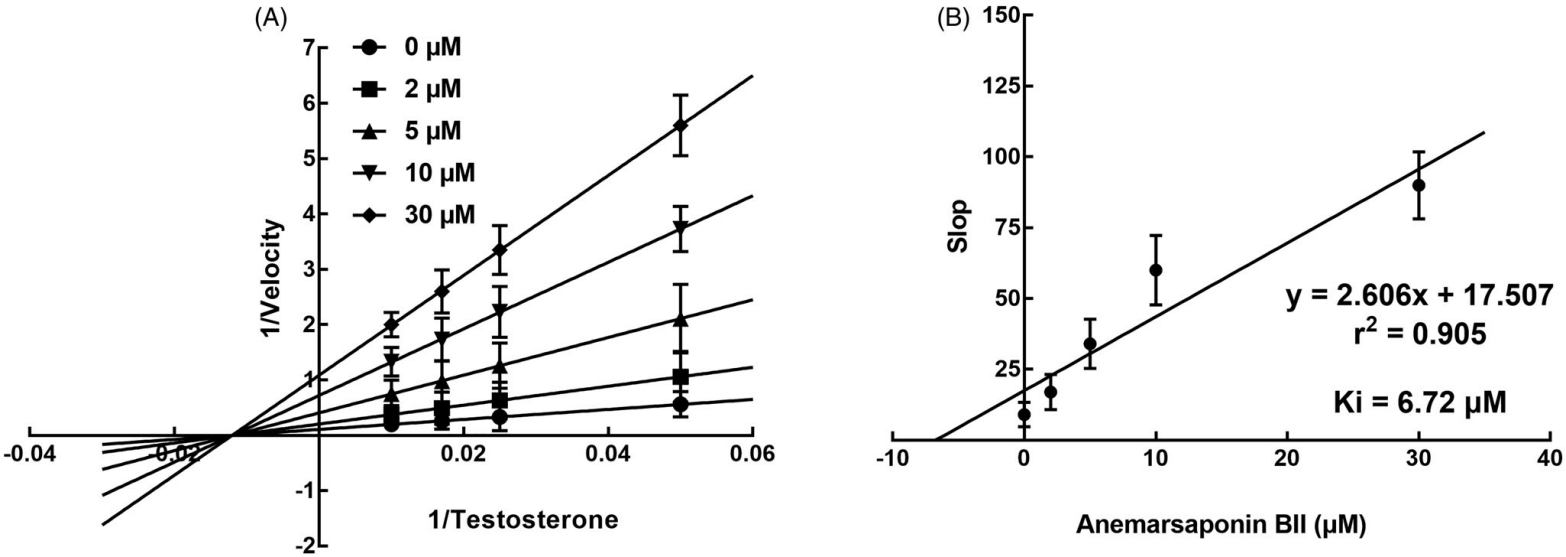
Lineweaver-Burk plots (A) and the secondary plot for *K_I_* (B) of inhibition of anemarsaponin BII on CYP3A4 catalysed reactions (testosterone 6β-hydroxylation) in pooled HLM. Data are obtained from a 30 min incubation with testosterone (20–100 μM) in the absence or presence of succinic acid (0–30 μM). All data represent the mean of the incubations (performed in triplicate).

**Figure 4.**
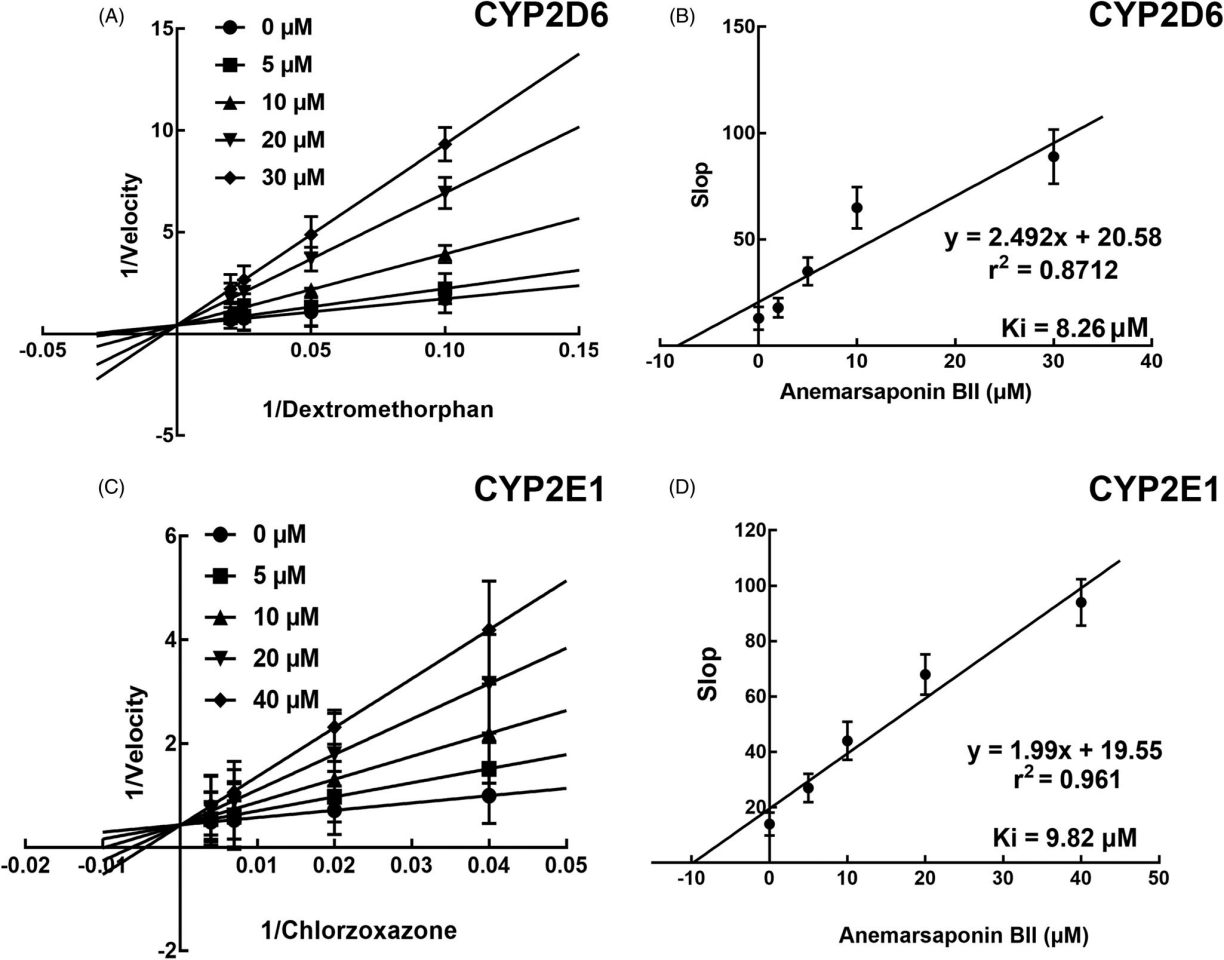
Lineweaver–Burk plots (A, C) and the secondary plot for *K_I_* (B, D) of inhibition of anemarsaponin BII on CYP2D6 catalysed reactions (dextromethorphan O-demethylation) and CYP2E1 catalyzed reactions (chlorzoxazone 6-hydroxylation) in pooled HLM. All data represent the mean of the incubations (performed in triplicate).

The Lineweaver–Burk transformation of the enzyme velocities substrates concentrations indicated that the inhibition of CYP3A4 by anemarsaponin BII was fitted in a non-competitive manner (Figure 3(A)). Moreover, the *K_I_* value of CYP3A4 was 6.72 μM (Figure 3(B)). For the inhibition of CYP2D6 and 2E1, anemarsaponin BII acted as a competitive inhibitor with the *K_I_* values of 8.26 and 9.82 μM, respectively (Figure 4(A–D)).

### Time-dependent manner of anemarsaponin BII in the inhibition of CYP3A4, 2D6 and 2E1

**Figure 5.**
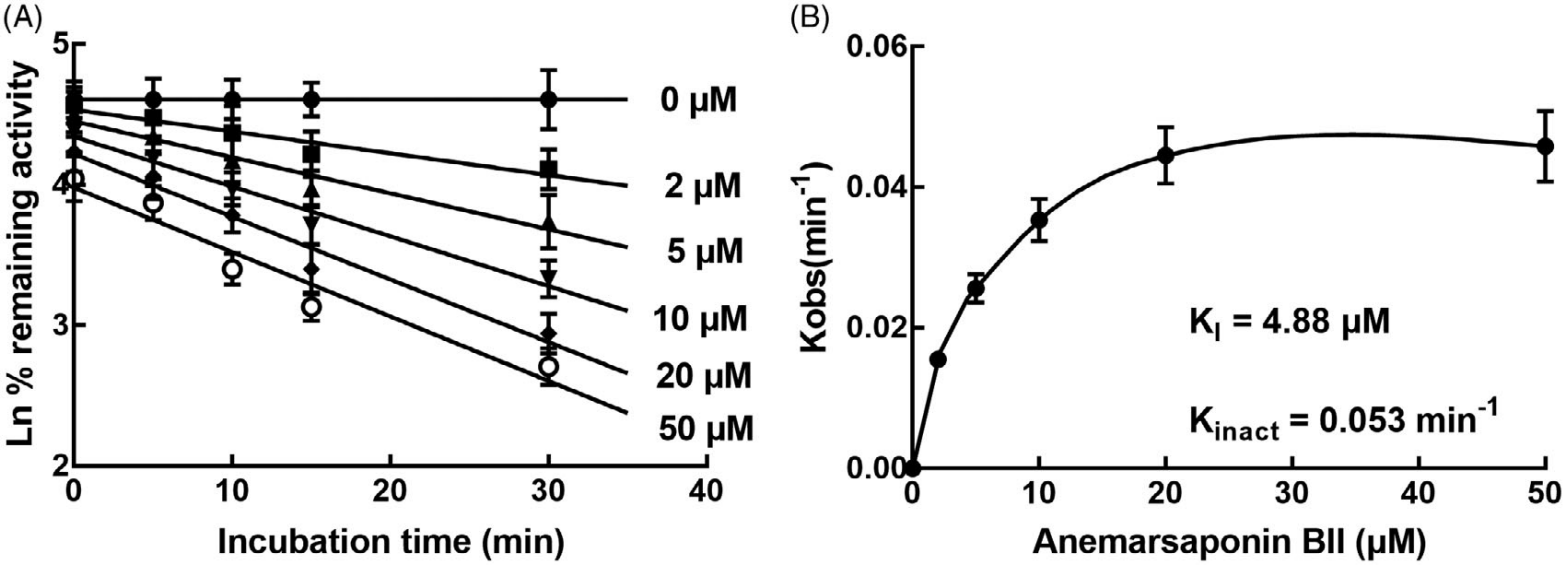
Time and concentration-inactivation of microsomal CYP3A4 activity by anemarsaponin BII in the presence of NADPH. The initial rate constant of inactivation of CYP3A4 by each concentration (*K_obs_*) was determined through linear regression analysis of the natural logarithm of the percentage of remaining activity versus pre-incubation time (A). The *K_I_* and *K_inact_* values were determined through non-linear analysis of the *K_obs_* versus the anemarsaponin BII concentration (B).

In the presence of 20 μM anemarsaponin BII, the inhibition of CYP3A4 was increased with the incubation time, while the inhibition of CYP2D6 and 2E1 was not affected by the incubation time (data not shown). Additionally, the values of *K_I_* and *K_inact_* value were calculated with the non-linear regression analysis in HLM to further characterize the time-dependent inhibition of CYP3A4 by anemarsaponin BII. It was obtained that the value of *K_I_* was 4.88 μM and the *K_inact_* was 0.053 min^−1^ (Figure 5).

## Discussion

This study characterized the effects of anemarsaponin BII on the activity of eight isoforms of CYP450 enzymes (CYP1A2, 2A6, 3A4, 2C8, 2C9, 2C19, 2D6 and 2E1) in human liver microsomes, which are commonly involved in the biotransformation of clinical drugs.

CYP3A is the most abundant human liver CYP enzyme, which is not only involved in the biotransformation of many endogenous substances, such as fatty acids, bile acids, and vitamin D_4_, but also regulates the metabolism of the majority of clinical drugs (Furge and Guengerich 2006). Previously, a number of drugs have been identified to affect the activity of CYP3A family, such as macrolide antibiotics (e.g., clarithromycin, and erythromycin), anti-HIV agents (e.g., ritonavir and delavirdine), antidepressants (e.g., fluoxetine and fluvoxamine), calcium channel blockers (e.g., verapamil and diltiazem), steroids and their modulators (e.g., gestodene and mifepristone), and several herbal and dietary components (He et al. 1999; Zhao et al. 2007; Zhou 2008; Sager et al. 2014; Freise et al. 2018; Akiyama et al. 2019). Here, anemarsaponin BII was revealed to be a weak non-competitive inhibitor of CYP3A4 with the IC_50_ value of 13.67 μM and the *K_I_* value of 6.72 μM. Additionally, the inhibition of CYP3A4 by anemarsaponin BII was found to be time-dependent with the *K_I_* and *K_inact_* values of 4.88 μM and 0.053 min^−1^. The inhibition of CYP3A4 could cause unfavourable and long-lasting drug–drug interactions and probably fatal toxicity. The present results indicated that anemarsaponin BII might induce drug–drug interaction with drugs metabolized by CYP3A4 via inhibiting the activity of CYP3A4, and the incubation time is an essential factor during the interaction.

Except for CYP3A4, CYP2D6 and CYP2E1 account for the vast majority of drug metabolism. In previous studies, CYP2D6 was considered as a non-inducible CYP isoform, which is consistent with the inhibitory effect of anemarsaponin BII this study (Inui et al. 2013; Bosilkovska et al. 2014). Moreover, the inhibition of CYP2D6 by anemarsaponin BII was found to be dose-dependent and in a competitive manner with an IC_50_ value and *K_I_* value of 16.26 and 8.26 μM, respectively. Unlike the inhibition of CYP3A4, the inhibition of CYP2D6 was not affected by the incubation time. Similar in CYP2E1, anemarsaponin BII also exerted a dramatic inhibitory effect on the activity of CYP2E1 competitively and the inhibition was not affected by incubation time. This kind of inhibition may increase the concentration of co-administrated drugs or herbs in the body, resulting in the toxicity, failure of treatment, or other adverse effects.

Previous studies on the *in vivo* pharmacokinetic profiles of anemarsaponin BII found that after administration of 10 g/kg *A. asphodeloide* extracts containing 103.1 mg/kg anemarsaponin BII, the maximum plasma concentration (*C_max_*) of anemarsaponin BII reached to 239.5 ng/mL with the half-life (*t*_1/2_) of 2.7 h (Li et al. 2015). Another study reported the administration of 40 mg/mL *A. asphodeloide* extracts containing 171.61 mg/kg anemarsaponin BII. The *C_max_* of anemarsaponin BII was obtained as 271.22 ng/mL with the *t*_1/2_ of 3.21 h (Tang et al. 2015). These values were far from the IC_50_ values of CYP3A4, 2D6, and 2E1. Therefore, the *in vivo* effect of anemarsaponin BII on the activity of CYPs needs further validation.

Anemarsaponin BII is the major component of *A. asphodeloide*, a widely used herb in paediatric (Kim et al. 2009; Zhao et al. 2016). The inhibitory effects of anemarsaponin BII on the activity of CYP3A4, 2D6, and 2E1 indicated the potential drug–drug interactions and side effects. In fact, there are a variety of factors that are associated with the metabolism of various drugs. For instance, *P-gp* is an important protein that related to the transport of various drugs and UDP-glucuronosyltransferases were considered to mediate the biotransformation of several xenobiotics, of which the activity also induced changes in the plasma concentration of drugs (Liu 2019b, 2019a). Therefore, the activity of these proteins and enzymes in the presence of anemarsaponin BII should be further investigated.

Taken together, anemarsaponin BII acted as a non-competitive inhibitor of CYP3A4 and competitive inhibitors of CYP2D6 and 2E1. Moreover, the inhibition of CYP3A4 was affected by the concentration of anemarsaponin BII and the incubation time, while the inhibition of CYP2D6 and 2E1 was only affected by the concentration of anemarsaponin BII. These results indicated the potential drug–drug interaction between anemarsaponin BII and drugs metabolized by CYP3A4, 2D6, and 2E1, which needs further studies to validate.
